# Preoperative MRI to Improve Aesthetic Outcomes in Secondary Mastopexy Augmentation: A Step-by-Step Approach

**DOI:** 10.1093/asjof/ojac068

**Published:** 2022-08-18

**Authors:** Brian P Dickinson, Monica B Vu, Melvin Silverstein, Krupa P Prajapati, January Lopez, Ellin D Li, Neal Handel

**Affiliations:** Plastic and reconstructive surgeon in private practice, Newport Beach, CA, USA; Research assistants; Gross Family Foundation Endowed Chair in oncoplastic breast surgery; Research assistants; Director of breast imaging, Hoag Hospital Newport Beach Breast Center, Newport Beach, CA 92663, USA; Physician assistant at a plastic surgery private practice, Newport Beach, CA, USA; clinical professor, University of California Los Angeles Department of Plastic & Reconstructive Surgery, Beverly Hills, CA 90210, USA

## Abstract

**Background:**

Secondary mastopexy augmentation is challenging because of compromised blood supply to the nipple areola complex (NAC). The operating surgeon often relies on clinical judgment and may perform a more conservative elevation of the NAC to minimize the risk of nipple necrosis. Despite this, the danger of necrosis persists. In our experience, MRI with contrast has enhanced preoperative planning in both cosmetic and reconstructive cases.

**Objectives:**

The goals of this article are to describe our use of preoperative MRI in identifying the blood supply to the NAC, evaluating dermo glandular thickness, decreasing surgical complications, and improving outcomes in secondary mastopexy augmentation.

**Methods:**

A consecutive series of secondary mastopexy augmentation procedures performed in 2021 were reviewed. In each case, preoperative maximum intensity projection (MIP) and/or high-resolution T1-weighted contrast enhanced MRI imaging was reviewed to elucidate the blood supply to the NAC and quantify the dermo glandular thickness. The imaging was used to formulate the operative plan. Preoperative and postoperative photographs were compared.

**Results:**

Eight cases were performed, four of which were selected to demonstrate our method using breast MRI with contrast in step-by-step approach. Patient satisfaction was high. The NAC survived in all cases.

**Conclusions:**

Surgeons can utilize preoperative breast MRI for strategic operative planning when performing secondary mastopexy augmentation. Visualization of the blood supply to the NAC and dermo glandular flap thickness are vitally important when performing a more aggressive lift of the breast.

**Level of Evidence: 3:**

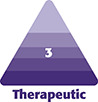

Reconstructive breast procedures following breast cancer surgery as well cosmetic breast augmentation are increasingly being performed. Approximately 1 in 8 women in the United States will develop breast cancer during their lifetime.^1^ In 2022, an estimated 287,850 new cases of invasive breast cancer were expected to be diagnosed in women in the United States, along with 51,400 new cases of noninvasive (in situ) breast cancer.^1^ In 2021, The Aesthetic Society demonstrated that there were 365,000 breast augmentations performed, and, in addition, 148,000 women had implants removed and replaced (+32% from 2020), and 71,000 had their implants removed and not replaced (+47%).^2^ The use of implants for breast reconstruction continues to be high with roughly 101,657 breast reconstruction procedures performed annually, of which 82% are implant-based (78,814 with silicone implants and 4402 with saline implants) and 18% were autologous tissue-based (18,441).^3,4^ The combination of breast implant procedures and oncoplastic reconstruction following lumpectomy create challenging scenarios in nipple areola complex (NAC) viability in secondary mastopexy procedures for plastic surgeons.

A literature review was conducted using PubMed and using the search terms, “secondary mastopexy,” “secondary mastopexy-augmentation,” “MRI,” “CT-scan,” and “CT-angiogram” in various combination which returned 0 results. Using “secondary mastopexy” and “secondary mastopexy augmentation” in combination with “imaging” and “ultrasound” resulted in one paper looking at the intraoperative use of “SPY angiography” to evaluate the insertion of saline implants of NAC blood flow prior to and after insertion of saline implants.^4^ Using the search terms “MRI” and “mastopexy,” one paper was identified that studied MRI prior to breast reduction surgery which determined that the predominant blood supply was to the NAC.^5^ Although inferior pedicle techniques were a common method for breast reduction, determining the location of the remaining blood supply for secondary mastopexy or mastopexy augmentation has not been described in the literature and is a challenging problem for plastic and reconstructive surgeons.

Challenges that a plastic and reconstructive surgeon may face include previous mastopexy, history of radiation, prior lumpectomy deformity, asymmetry, ptosis, and implant rupture. Secondary mastopexy augmentation is associated with increased risk of NAC necrosis and poor wound healing because of the blood supply being disrupted by a previous aesthetic or reconstructive procedure.^6,7^ A conservative lift of the NAC might be considered to minimize further disruption, but the inherent risk to the NAC remains. In our experience, the technique of MRI imaging to determine the blood supply to the NAC can help prevent such complications, as demonstrated by a step-by-step surgical planning method.

## METHODS

A **retrospective** chart review was conducted on consecutive patients with previous augmentation, mastopexy augmentation, or breast reconstruction who underwent mastopexy during 2021. Consent was obtained from all participants. In all, charts were reviewed and categorized by the most commonly presenting clinical scenarios and then analyzed by the most common challenges affecting revisional mastopexy. In all cases, preoperative MRI images of the patients’ breasts were obtained. Methods and maneuvers for identifying the vascular pedicle on the MRI imaging and creating a surgical plan were discussed. The preoperative and postoperative photos were analyzed. We purposefully selected between 1- and 2-week postoperative photographs to examine skin T-junction and NAC viability. Satisfaction was determined by patient response. We determined bra sizing for our patients as described by Pechter.^8^

## RESULTS

Eight consecutive patients underwent secondary mastopexy augmentation who had a preoperative MRI prior to their procedure. Five patients had a previous history of breast cancer treated with lumpectomy and radiation, and 3 patients underwent previous cosmetic breast augmentation or mastopexy augmentation. The age range was 43-77 years. No patients were current smokers. No patient had a history of diabetes. Six of the 8 patients had a healthy body mass index (BMI), and 2 patients had a BMI in the overweight category (> 25).

Four patients were selected because case studies that represented and illustrated the most common clinical problems we encountered where we utilized the MRI to help formulate our surgical plan. The first patient had a history of lumpectomy, mastopexy, and radiation therapy, and the second patient previously underwent a cosmetic mastopexy augmentation procedure. The third patient wanted explanation of her implants and a lift who underwent 3 previous augmentations and a breast reduction. Finally, a fourth patient who underwent correction of bilateral mastectomy reconstruction was presented for instructive comparison using the preoperative MRI.

### Case 1: Previous Breast Cancer and Radiation Therapy

#### History

The patient is a 73-year-old female with a history of right breast cancer treated with lumpectomy and radiation therapy 20 years prior. She had subsequently undergone insertion of right and left breast mammary prosthesis with a left mastopexy for symmetry a year after her radiation therapy. She developed a right grade IV capsular contracture and had breast asymmetry.

The patient was not aware of her exact bra size because she only wore sports bras because of her asymmetric breasts. She complained of pain and being able to feel the edges of her implant through her skin, and she was dissatisfied with her breast asymmetry. Her goals were to be larger in size and to have her asymmetry corrected.

#### Objective

The patient’s current bra size was unknown, and she desired to be a 34 C postoperatively. Her measured chest circumference was 29.5 inches, her right breast cup-size was 8 inches, and her left breast cup-size was 9 inches. This made her right breast a 34 B/C and her left breast a 34 C/D. The right sternal notch to nipple distance was 22 cm, and the left breast was 23 cm. The right nipple to fold distance was 9.5 cm and the left nipple to fold distance was 11 cm. The right and left breast base diameters were 11 cm. The right breast had a Baker IV capsular contracture.

The right breast had a scar in the upper inner quadrant of the breast from her previous lumpectomy. She had loss of volume and concavity beneath the lumpectomy scar. The patient had scars in the inframammary fold and circumareolar mastopexy scars bilaterally. The implants were in the retropectoral position.

#### Assessment

The patient had multiple previous scars on the breast which could potentially impede arterial inflow or venous outflow to the NAC, and this presented a significant challenge. There were no prior operative reports available. We proceeded with the following steps to help formulate our surgical plan:

**Step 1:** A breast MRI with contrast was ordered to evaluate the blood supply to the NAC (Video 1).

**Figure 1.**
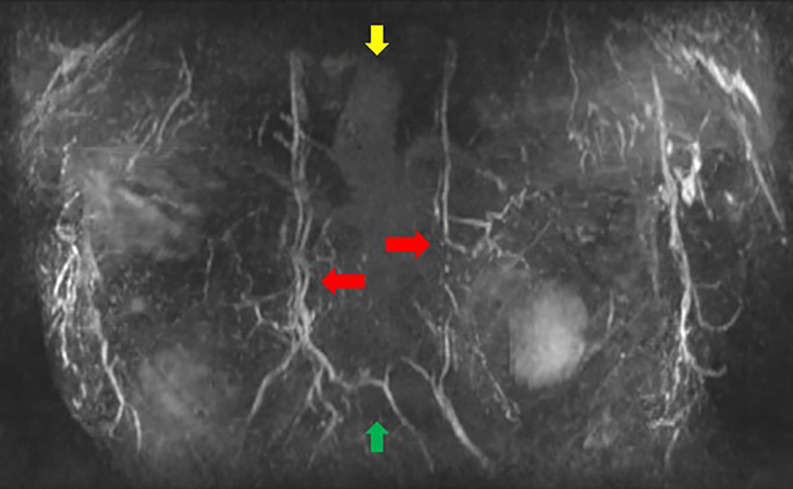
Coronal MRI maximum intensity projection (MIP) images. The manubrium of the sternum is at the top of the image (yellow arrow), and the xiphoid is at the bottom of the image (green arrow). The internal mammary vessels can be seen coursing on either side of the sternum with pectoralis major perforators originating from the internal mammary vessels (red arrows).

**Figure 2.**
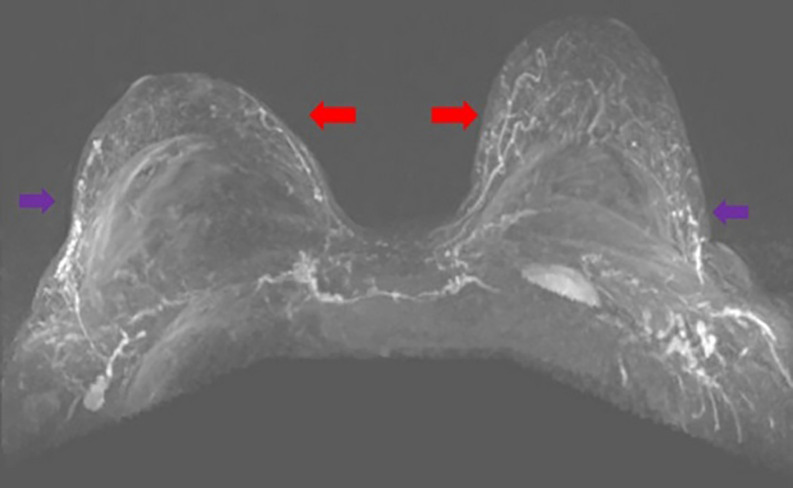
Axial MRI maximum intensity projection (MIP) images. The pectoralis major perforators originating from the internal mammary vessels (red arrows). The vessels can be traced heading toward the nipple areola complex. The lateral vessels (purple arrows) do not quite reach the nipple areola complex on either side. Therefore, in this case, the medial blood supply was selected to keep the nipple areola complex alive.

**Step 2:** The MIP coronal images (Figure 1) and axial images (Figure 2) were evaluated to determine if the predominant blood supply was originating from the medial and/or lateral aspect of the breast. Reviewing the MIP subimages in series allowed for visualization of the dominant blood supply to the NAC originating from superior medial perforators.

**Figure 3.**
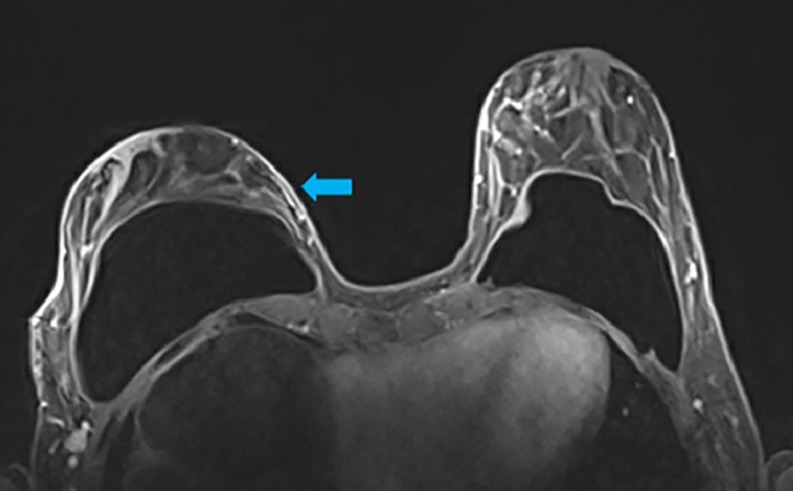
The axial high-resolution images allow individual perforating vessels to be traced along their course (blue arrow). At the location of the blue arrow, the dermoglandular thickness was 1.4 cm. Therefore, the medial blood supply was selected to keep the nipple areola complex alive, and undermining would be limited in this location and more deepithelialization would be done to maintain this blood supply.

**Step 3:** The high-resolution views were evaluated next (Video 2). The high-resolution further delineates the course of the blood supply to the NAC as well as to determine the plane of the implant and dermo glandular thickness (Figure 3). Additionally, there was no evidence of breast cancer recurrence.

**Step 4:** The plan:

1. Decrease the implant size to reduce capsular contracture risk recurrence and prevent tension on the wound edges. Maintain the superior medial vascular blood supply to the NAC and gain lateral access to the right breast implant capsule to prevent disrupting the NAC blood supply. Maintain the right mammary prosthesis in the retropectoral position.
2. Right breast selective capsulotomy/capsulectomy to lateralize the right breast. Right breast Wise pattern mastopexy to reduce NAC diameter and cautious deepithelialization of the pattern to facilitate venous drainage in all directions.
3. Revise the right breast lumpectomy scar to correct concavity after the Wise pattern mastopexy and implant exchange and viability of NAC has been confirmed.
4. Left mastopexy/reduction and insert smaller implant exchange through a Wise pattern mastopexy. Maintain the left mammary prosthesis in the retropectoral position.

#### Postoperative Results

**Figure 4.**
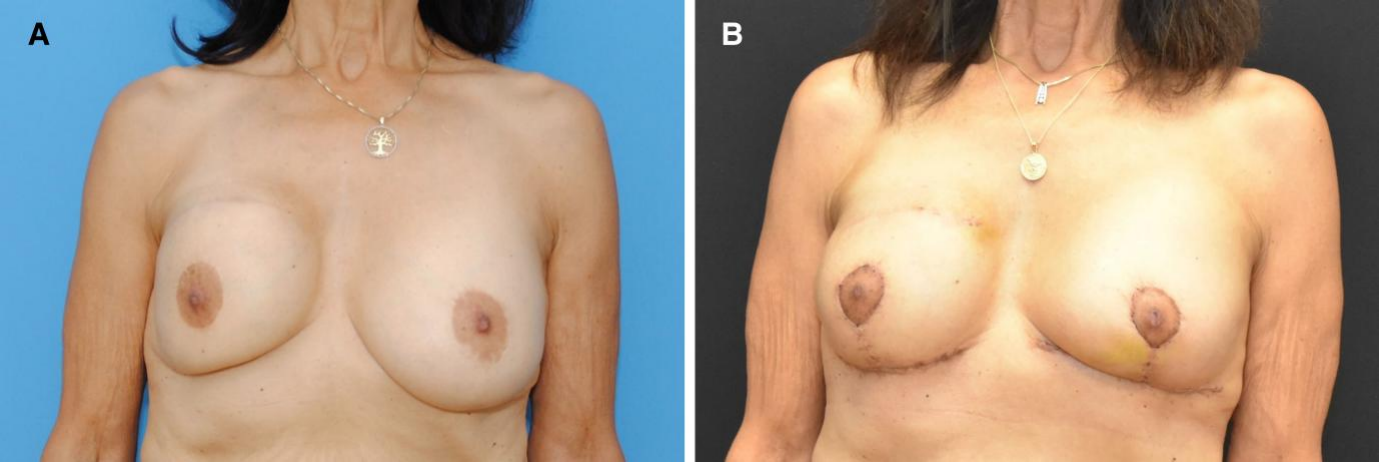
A 73-year-old female (A) with previous bilateral circumareolar mastopexy scars, bilateral inframammary fold scars, and previous right breast lumpectomy and radiation. The multiple previous incisions made it challenging to predict how to maintain viability of the nipple. (B) Two weeks postoperatively, the nipple areola complexes were viable bilaterally. There was no evidence of delayed healing at the T-junction. Preoperative and postoperative comparison of the surgical outcome displayed an excellent surgical result.

Postoperatively, the patient was satisfied with the aesthetic result and maintained the blood supply to the bilateral NACs. There was no delayed healing at the T-junction of the Wise pattern mastopexy. The patient was satisfied with the aesthetic result and maintained the blood supply to the bilateral NACs. There was no delayed healing at the T-junction of the Wise pattern mastopexy (Figure 4).

### Case 2: Previous Bilateral Mastopexy Augmentation

#### History

Patient was a 49-year-old female with a history of bilateral augmentation mammoplasty and bilateral mastopexy. She had saline implants filled to 575 cc bilaterally. She presented to discuss her options for secondary breast augmentation and mastopexy.

The patient was unhappy with the size and shape of her breasts, expressing they were too large and pendulous. She also wished to decrease the size of NAC.

#### Objective

The patients self-reported bra size was a 38 DD/DD, and she wished to decrease her size to a 38 C postoperatively. Measurement of her chest circumference was 33 inches with the right cup-size measuring 11 inches and the left cup size measuring 12 inches. The resulting bra size was a 38 D/DD on the right and a 38 DDD on the left. The right sternal notch to nipple distance was 26 cm, and the left sternal notch to nipple distance was 26 cm. The right nipple to fold distance was 17 cm, and the left nipple to fold was 17 cm. The breasts were a grade II ptosis bilaterally. The implants were in the prepectoral position bilaterally.

#### Assessment

The patient had bilateral circumareolar scars. She had a sternal notch to nipple distance of 26 cm bilaterally. The dermo glandular flaps were thin, with the implant easily palpable and visible beneath the skin. There was no prior operative report available. We proceeded with the following steps to help formulate our surgical plan:

**Step 1:** A breast MRI with contrast was ordered to evaluate the blood supply to the NAC (Video 3).

**Figure 5.**
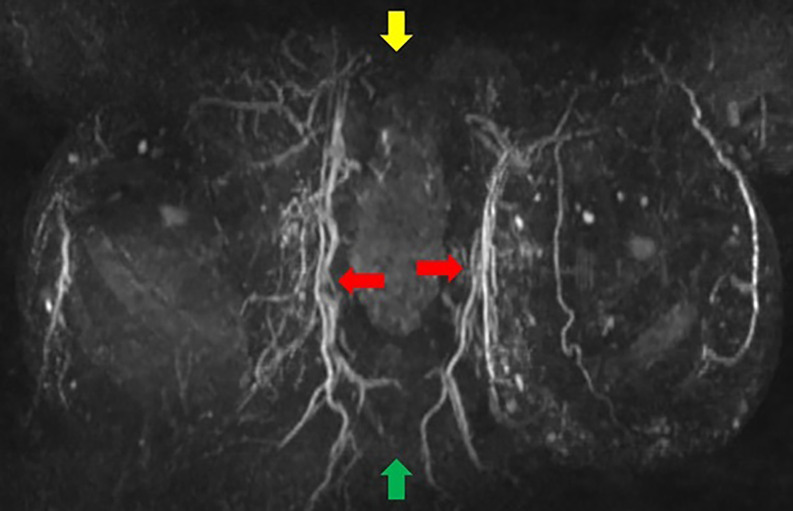
Coronal MRI maximum intensity projection (MIP) images. The manubrium of the sternum is at the top of the image (yellow arrow), and the xiphoid is at the bottom of the image (green arrow). The internal mammary vessels can be seen coursing on either side of the sternum with pectoralis major perforators originating from the internal mammary vessels (red arrows).

**Figure 6.**
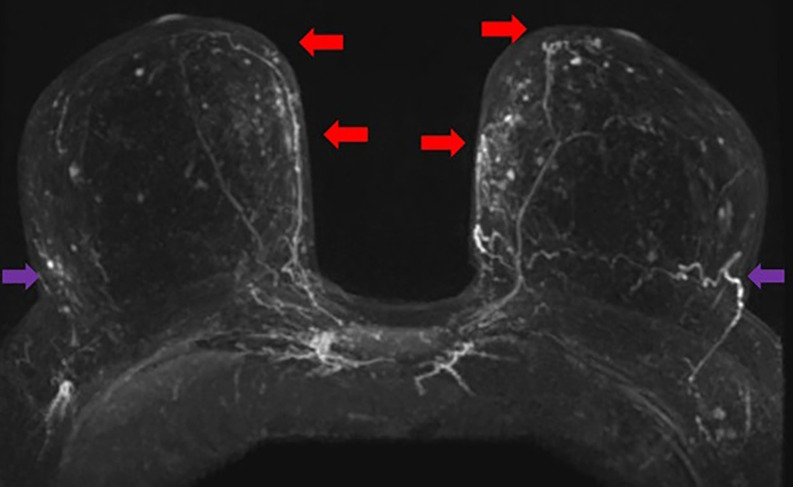
Axial MRI maximum intensity projection (MIP) images. The pectoralis major perforators originating from the internal mammary vessels (red arrows). The vessels can be traced heading toward the nipple areola complex. The lateral vessels (purple arrows) do not quite reach the nipple areola complex on either side. Therefore, in this case, the medial blood supply was selected to keep the nipple areola complex alive.

**Step 2:** The MIP images, coronal images (Figure 5), and axial images (Figure 6) were evaluated to determine if the predominant blood supply was originating from the medial and/or lateral aspect of the breast. The preoperative MRI series indicated a dominant superior medial-based blood supply.

**Figure 7.**
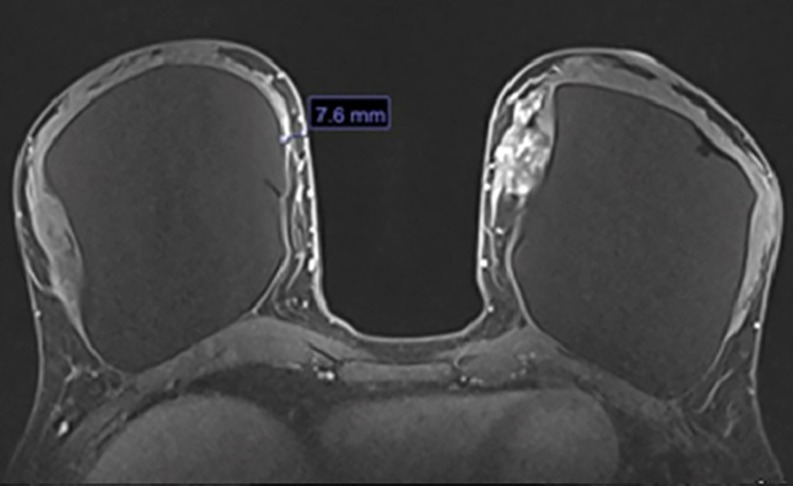
The axial high-resolution images allow individual perforating vessels to be traced along their course. At the location, the dermoglandular thickness was 7.6 mm. Therefore, the medial blood supply was selected to keep the nipple areola complex alive, and undermining would be limited in this location and more deepithelialization would be done to maintain this blood supply. The suspicious lesion of the left breast was biopsied and determined to be benign. The mammary prosthesis was located in the subglandular plane.

**Step 3:** The high-resolution views were examined next (Video 4). The high-resolution views further delineate and visualize the course of the blood supply to the NAC, determine the plane of the implant, and evaluate dermoglandular thickness (Figure 7). There was a suspicious lesion in the left breast which was subsequently biopsied and determined to be benign. The patient was recommended to repeat imaging of the breast in 6 months for surveillance.

**Step 4:** The plan:

1. Remove the bilateral mammary prosthesis and replace with smaller mammary prosthesis. Lateral access to the breast implants bilaterally to prevent disrupting the NAC blood supply.
2. Selective capsulotomy/capsulectomy to allow expansion of the breast pocket while leaving a small supportive strip inferiorly to prevent bottoming out.
3. Maintain the mammary prosthesis in the subglandular plane.
4. Bilateral mastopexy maintains a superior medial blood supply to the NAC.
5. Goal breast diameter size: 9.5 inches.

#### Postoperative Results

**Figure 8.**
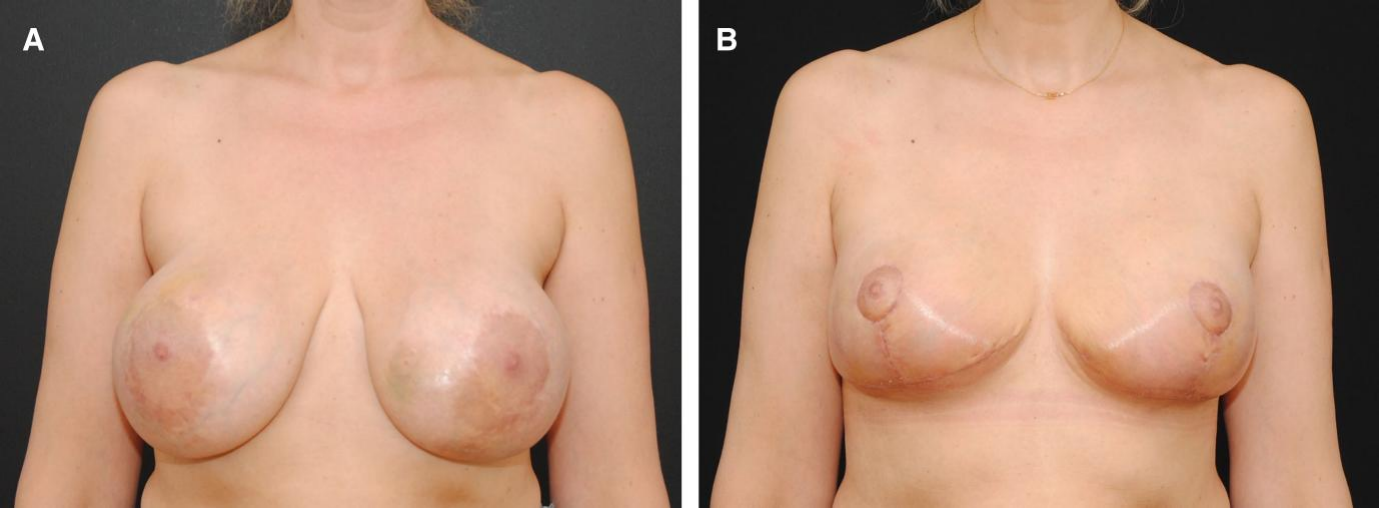
A 49-year-old female (A) with previous circumareolar mastopexy augmentation. The patient wanted to reduce the size of her nipple areola complex as well as significantly lift the breasts. The implants could be easily seen and palpated beneath the skin. It was challenging to ascertain the blood supply to the nipple areola complex for a significant lift and change. (B) Postoperative comparison of the surgical outcome at 2 weeks, the nipple areola complexes were viable bilaterally. There was no evidence of delayed healing at the T-junction.

There was a satisfactory elevation of the NAC and reduction of the size of the areola. The patient was happy with her results (Figure 8).

### Case 3: Lateral Pedicle Dominance

A 49-year-old patient with 3 previous bilateral augmentation mammoplasty procedures and 1 previous bilateral mastopexy wished to undergo removal of her mammary prosthesis, breast reduction, and decrease the size of her NAC. She had saline implants filled to 200 cc bilaterally.

#### Objective

The patient’s self-reported bra size is a 34 DD, and she wished to be a 34 B following revision if possible and to decrease the size of her NAC. Her chest circumference was 32 inches, and the right breast cup size was 11 inches, and the left breast cup size was 11 inches, making her bra size a 36 DD bilaterally. The right sternal notch to nipple distance was 23 cm, and the left sternal notch to nipple distance as 23 cm. The right nipple to fold was 14 cm, and the left nipple to fold was 14 cm. The breasts had glandular ptosis bilaterally, and the nipple required moderate elevation.

#### Assessment

The patient had bilateral Wise pattern mastopexy scars bilaterally. She had a sternal notch to nipple distance of 23 cm bilaterally. The dermo glandular flaps were thick. There was no prior operative report available. We proceeded with the following steps to help formulate our surgical plan:

**Step 1:** A breast MRI with contrast was ordered to evaluate the blood supply to the NAC given the history of 3 previous breast augmentations and previous mastopexy.

**Figure 9.**
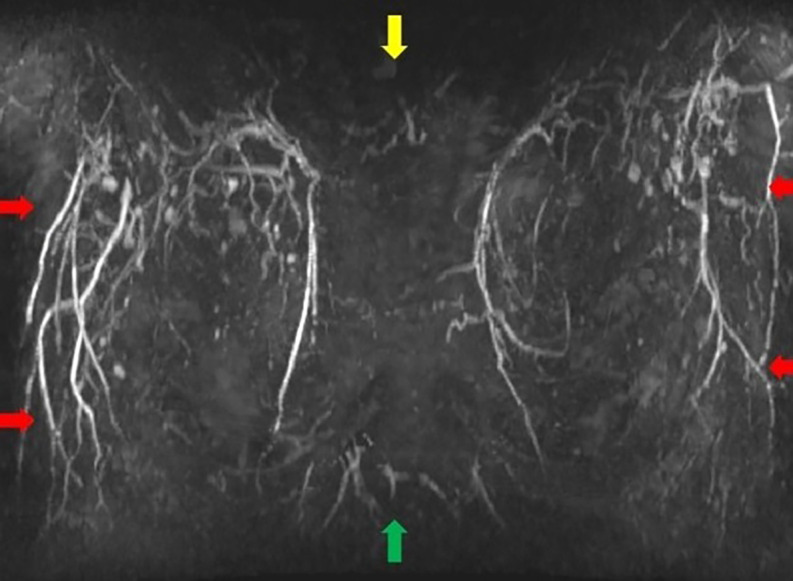
Coronal MRI maximum intensity projection (MIP) images. The manubrium of the sternum is at the top of the image (yellow arrow), and the xiphoid is at the bottom of the image (green arrow). The lateral mammary vessels are more dominant and robust than the perforators arising from the internal mammary vessels (red arrows).

**Figure 10.**
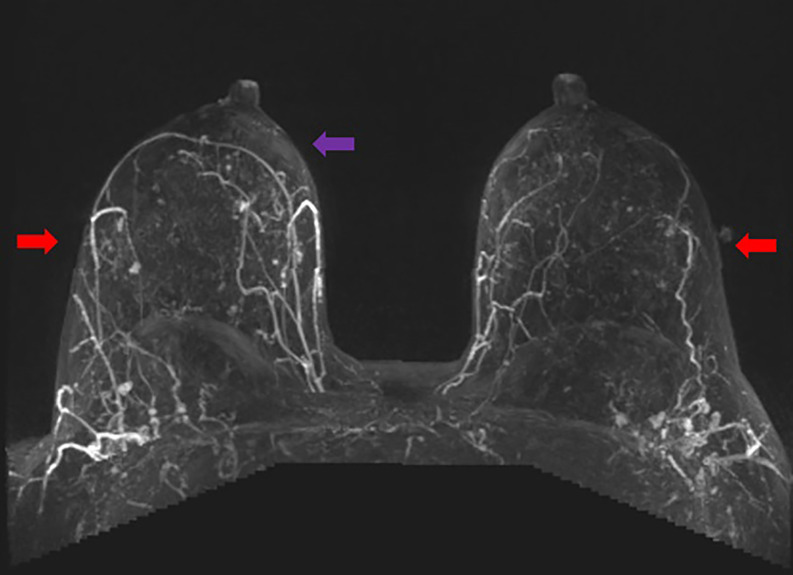
Axial MRI maximum intensity projection (MIP) images. The lateral mammary vessels are more dominant (red arrows) and extend further to the nipple areola complex than the medial perforators off the internal mammary vessels (purple arrows) which do not quite reach the nipple areola complex on either side. Therefore, in this case, the lateral blood supply was selected to keep the nipple areola complex alive.

**Step 2:** The MIP images were evaluated by examining the series of images. The coronal images are evaluated first (Figure 9), and the axial images were evaluated second (Figure 10) to determine if the predominant blood supply was originating from the medial or lateral aspect of the breast or both. The preoperative MRI series showed dominant lateral based blood supply bilaterally.

**Figure 11.**
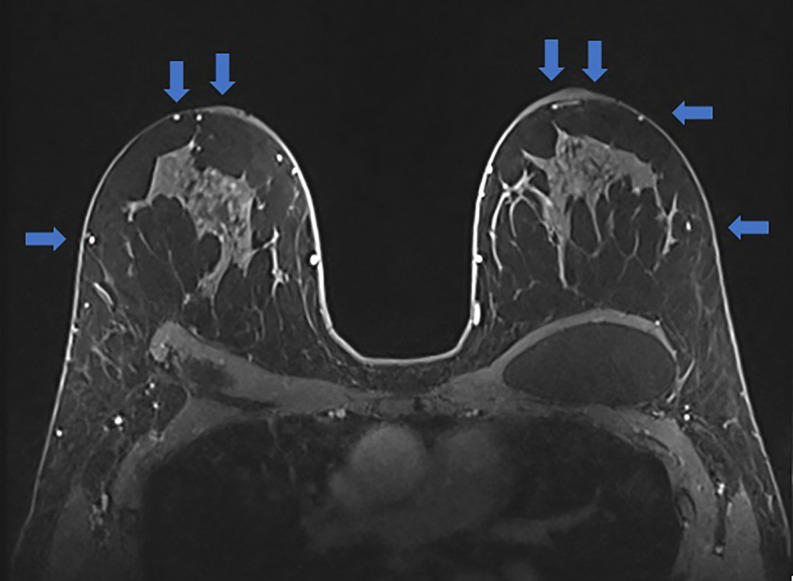
The axial high-resolution images allow individual perforating vessels to be traced along their course (blue arrow). At the location of the blue arrow, the blood vessels arising from the lateral mammary vessels could be traced to course in closest proximity to the nipple areola complex. Therefore, the lateral blood supply was selected to keep the nipple areola complex alive, and undermining would be limited in this location and more deepithelialization would be done to maintain this blood supply. The medial blood vessels could not be traced into close proximity of the nipple areola complex.

**Step 3:** The high-resolution views were examined to further delineate and visualize the course of the blood supply to the NAC as well as determine the plane of the implant, and dermoglandular thickness (Figure 11). Tracing the lateral based blood supply and arcade using the high-resolution images demonstrated that the NAC was supplied by the lateral vascular arcade. The medial pedicle did not course as proximally to the NAC as the lateral pedicle, and on the left breast, the NAC arcade was supplied predominantly by the lateral vascular pedicle.

**Step 4:** The plan:

1. Removal bilateral mammary prosthesis. Inferior access to the breast implants in the prepectoral plane and removal bilaterally to prevent disrupting the NAC blood supply.
2. Selective capsulotomy/capsulectomy to allow healing of the breast pocket and seroma prevention.
3. Bilateral mastopexy keeping a superolateral blood supply to the NAC and undermining more medially for closure and redraping.
4. Goal breast diameter size: 8 inches.

#### Postoperative Results

**Figure 12.**
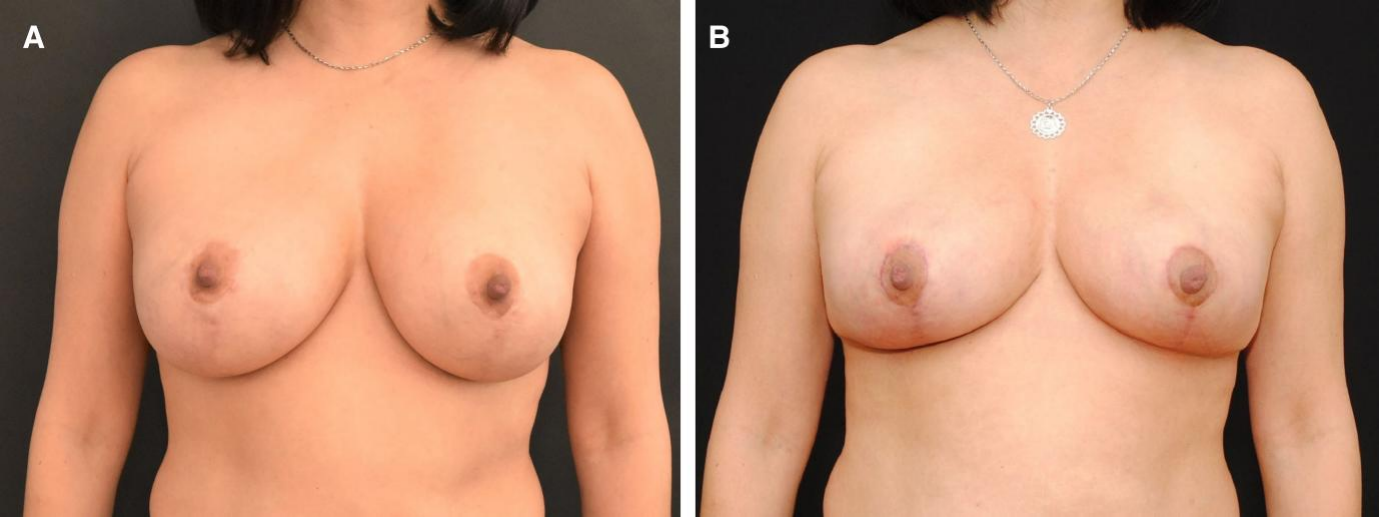
A 49-year-old female (A) with 3 previous augmentation mammoplasty procedures and 1 previous bilateral mastopexy. The patient wanted to reduce the size of her nipple areola, remove her mammary prosthesis, and moderate lift the breasts. It was challenging to ascertain the blood supply to the nipple areola complex given the multiple previous operations. (B) Postoperative comparison of the surgical outcome at 2 weeks, the nipple areola complexes were viable bilaterally. There was no evidence of delayed healing at the T-junction.

There was a satisfactory elevation of the NAC, and the NAC remained viable. The patient was happy with her results (Figure 12).

### Case 4: Post Mastectomy Reconstruction MRI

A 60-year-old patient who is post bilateral nipple sparing mastectomies with tissue expander and implant-based breast reconstruction. She had undergone revision of her reconstruction in the past as well as excision of a local breast cancer recurrence. She wished to undergo removal of her mammary prosthesis and replacement to a smaller size and undergo a lift.

#### Objective

The patient’s self-reported bra size is a 38 C, and she wished to be a 38 B following revision and to decrease the size of her NAC. The measured chest circumference was 32 inches with a right breast cup size of 9 inches and a left breast cup size of 9 inches for a bra size of 36 DD bilaterally. The right sternal notch to nipple distance of 20.5 cm, and the left sternal notch to nipple distance was 21 cm. The right nipple to fold distance was 10 cm, and the left nipple to fold distance was 10 cm. The breasts were not ptotic and required minimal nipple elevation.

#### Assessment

The patient had superior bilateral batwing mastectomy scars bilaterally (Figure 16). She had a sternal notch to nipple distance of 21 cm bilaterally. The dermo glandular flaps were thin given her previous mastectomy. There was no prior operative report available.

**Step 1:** A breast MRI with contrast was ordered to evaluate the blood supply to the NAC given the history of bilateral mastectomy and bilateral batwing incisions.

**Figure 13.**
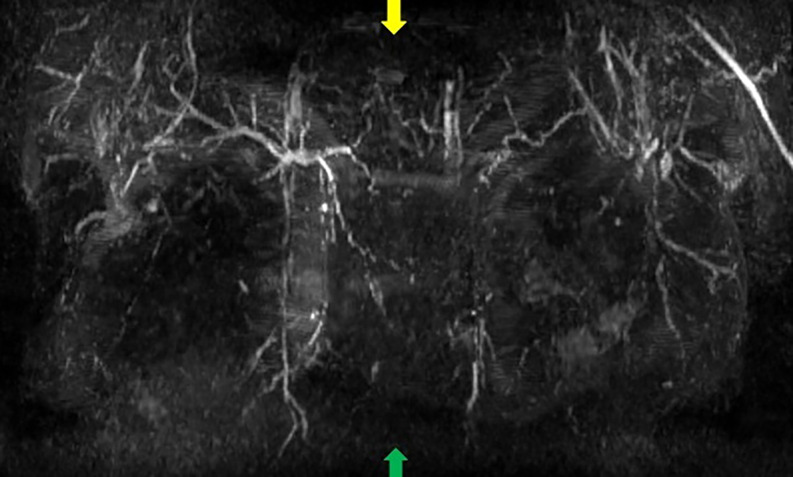
Coronal MRI maximum intensity projection (MIP) images. The manubrium of the sternum is at the top of the image (yellow arrow), and the xiphoid is at the bottom of the image (green arrow). There is an absence of robust perforators from neither lateral mammary vessels nor pectoralis perforators off the internal mammary vessels.

**Step 2:** The MIP images were evaluated by examining the series of images. The coronal images are evaluated first (Figure 13) which showed minimal to no direct axial perfusion of the NAC. The left breast had predominantly lateral perfusion, and the right breast had predominantly medial perfusion.

**Figure 14.**
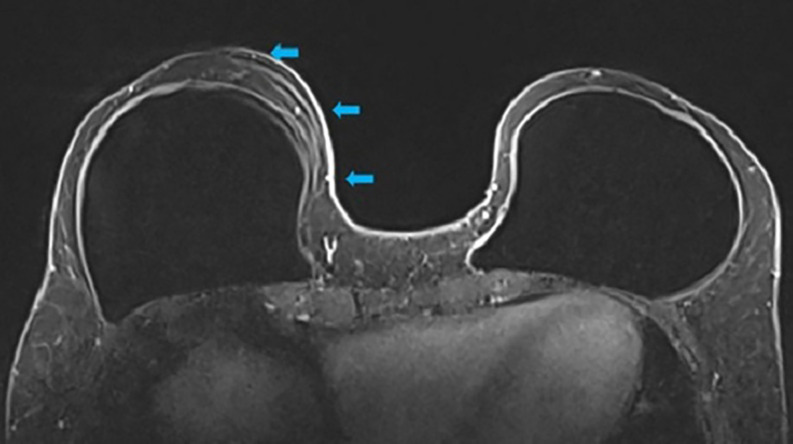
The axial high-resolution images allow individual perforating vessels to be traced along their course (blue arrow). At the location of the blue arrow, the blood vessels arising from the internal mammary vessels could be traced to course in closest proximity to the nipple areola complex. Therefore, the medial blood supply was selected to keep the nipple areola complex alive, and undermining would be limited in this location and more deepithelialization would be done to maintain this blood supply.

**Step 3:** The high-resolution views were examined to further delineate and visualize the course of the blood supply to the NAC as well as to determine the plane of the implant and dermoglandular thickness (Figure 14). On the right, tracing the medial-based blood supply and arcade using the high-resolution images demonstrated that the NAC was supplied by the medial vascular arcade superior to the NAC. This was correlated with the presence of a superior batwing crescent excision.

**Step 4:** The plan:

1. Removal of bilateral mammary prosthesis. Inferior lateral access to the breast implants and removal bilaterally to prevent disrupting the NAC blood supply. Strict circumareolar deepithelialization and deepithelialization of inferior dermoglandular pennant to facilitate venous drainage.
2. Selective capsulotomy/capsulectomy to allow healing of the breast pocket and seroma prevention.
3. Bilateral mastopexy keeping a superolateral blood supply to the NAC and undermining more medially for closure and redraping.
4. Goal breast diameter size: 8 inches.

#### Postoperative Results

**Figure 15.**
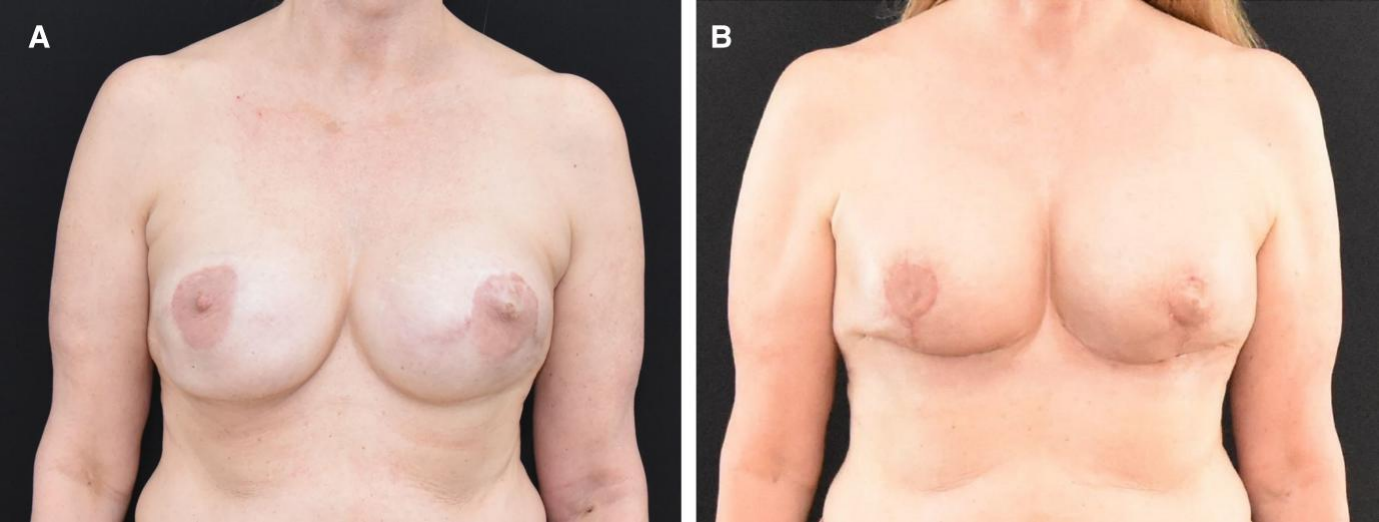
A 60-year-old female patient (A) who is post bilateral nipple sparing mastectomies with tissue implant-based breast reconstruction. She had undergone revision of her reconstruction in the past as well as excision of a local breast cancer recurrence. She wished to undergo removal of her mammary prosthesis and replacement to a smaller size and undergo a lift. (B) Preoperative and postoperative comparison of the surgical outcome at 2 weeks, the nipple areola complexes were viable bilaterally. There was no evidence of delayed healing at the T-junction.

There was a satisfactory elevation of the NAC, and the NAC remained viable with no evidence of delayed healing. The patient was happy with her results (Figure 15).

## DISCUSSION

The case studies elucidate the utility of preoperative MRI breast imaging in optimizing outcomes following revisional mastopexy for cosmetic patients as well as those with a history of breast cancer. Preoperative MRI can yield significant information as follows: (1) detect occult cancers, (2) identify implant rupture, (3) determine the remaining blood supply to the NAC, and (4) determine the dermo glandular thickness to limit undermining in those areas. This information is invaluable to aesthetic and reconstructive breast surgeons in planning secondary mastopexy or mastopexy-augmentation operations. The data provided by MRI can improve outcomes and limit complications.

Often, resection of the NAC is completed when malignant tumors are close to the nipple.^9^ With proper planning, the skin patterns chosen can facilitate favorable wound closure and healing and result in aesthetically pleasing breast mounds. Unintended loss of the NAC in aesthetic or reconstructive procedures is a devastating complication for the patient and the operating surgeon. Unanticipated NAC necrosis creates a prolonged healing process by secondary intention that involves frequent dressing changes, soiling on the bra, and emotional stress on the patient. Often there is significant underlying fat necrosis that hinders the ultimate cosmetic result. Having to undergo debridement and daily wound care is often time consuming and frustrating for both the patient and the physician. Breast MRI with contrast combined with excellent interpretation of the maximum intensity projection (MIP) images and high-resolution images can minimize complication and improve patient outcomes and satisfaction. Invariably, this will improve physician satisfaction.

In the aesthetic secondary mastopexy-augmentation patient, the preoperative MRI is also beneficial to evaluate for breast cancer, implant integrity, and a great adjunct for preoperative planning and execution. Although not a mandatory prerequisite, it is recommended in complex mastopexy cases. Although one can assume that the dominant blood supply to the NAC is from the superior medial intercostal vessels, to make this assumption in the secondary mastopexy or secondary mastopexy-augmentation patient can result in significant patient morbidity if major lift or implant volumes are added in the subglandular position. Once we identify the predominant or remaining blood supply on the preoperative MRI, we then draw out the pedicle in the preoperative marking area or in the operating room to remind ourselves not to dissect or raise skin flaps in this area. Occasionally, the handheld Doppler is used to map out vessels in the operating room in the position that we are going to operate. It is important to remember that when a patient is undergoing an MRI, they are in the prone position. When marking in the preoperative area, the patient is standing, and when marking on the operating room table, the patient is in the supine position. Taking note of these different positions can make one more comfortable marking over time.

In the third patient with multiple previous operations, the dominant remaining blood supply was provided by a lateral pedicle. One should use caution assuming the nipple can survive on purely random blood supply when performing major nipple elevation or adding an implant with volume, and steps should be taken, when possible, to identify blood supply location. In mastopexy-augmentation, skin flaps need to be developed to allow re-draping of the skin around the NAC. When extensive undermining is performed, maintaining a random pattern blood supply is not possible or becomes extremely limited by the undermining. Selecting a discrete/dominant pedicle in these instances can allow for greater mobilization of the NAC as well as re-draping of the skin. Great care should be taken to preserve the pedicle serving the NAC, and one should limit undermining in this location. After evaluating the MIP images of the MRI to determine NAC blood supply, the surgeon should review the high-resolution images to trace these blood vessels to the NAC the best they can and limit surgical undermining in this region.

Some surgeons may criticize MRI in the aesthetic population as an unnecessary cost. We have found this not true. In challenging cases, without the MRI, patients may need to undergo a 2-stage operation. Stage 1 would include removal of implants and mastopexy. Stage 2 would then be insertion of mammary prostheses. The cost of two operations could be much more than the cost of an MRI. Furthermore, nipple necrosis or significant fat necrosis debridement can dramatically increase the cost. Costs will continue to rise if multiple debridements are required following surgery to correct a less than optimal result.

It is prudent to discuss insurance authorization for MRI prior to secondary mastopexy augmentation. Many patients presenting for secondary mastopexy augmentation are of an age when mammographic screening is recommended. If there is an insurance authorization obstacle, we try to convey in the peer-to-peer review process that the MRI will help detect breast cancer, evaluate implant integrity, and allow for an appropriate evaluation of the NAC blood supply. An MRI often affects the surgical plan and oncologic treatment. A mammogram and an ultrasound of the breast cannot evaluate the blood supply to the NAC and is unreliable for assessing implant integrity. Both knowing how to preserve the NAC and the condition of the implant are important to the operating surgeon. In patients with cancer, MRI is often needed in addition to a mammogram for complete screening. In addition to evaluating cancer and blood supply to the NAC, the MRI also yields information pertaining to the plane of the mammary prosthesis as well as the thickness of the skin and breast flap above the mammary prosthesis.

The average cost for a breast MRI ranges from $250 to $700 USD in the United States. Therefore, it is up to the surgeon and patient to discuss the cost of an MRI compared with risks and benefits of not obtaining an MRI. One patient in our study received an out-of-town MRI for $2000 USD. This is not an unreasonable cost in a patient where the operating surgeon has no idea of the remaining blood supply to the NAC. MRI review can not only prevent NAC ischemia and fat necrosis, but it is also often the patients only encounter with healthcare personnel able to detect early breast cancer and high-risk lesions. The benefit is priceless. Investment in an MRI can prevent NAC ischemia and fat necrosis, thereby preventing one or more return trips to the operating room which could exceed the cost of the MRI in locations where an MRI is more affordable. Although MRI screening is commonplace for our patients with breast cancer, we also strongly recommend MRI in those women undergoing secondary mastopexy who have had multiple prior operations and/or thin dermoglandular flaps with implants in the subglandular position. The cost and benefit of completing a preoperative MRI should be discussed with the patient along with the inherent risks of secondary mastopexy augmentation.

Once the MRI is obtained, the operating surgeon should review it to determine if there are any suspicious lesions that need to be addressed prior to embarking on aesthetic or cancer reconstruction. Suspicious lesions are coordinated through a comprehensive breast center for core needle biopsy. If the lesion is benign and concordant with the MRI findings, the reconstructive surgeon can proceed with secondary mastopexy, mastopexy-augmentation, or reduction mammoplasty. If the lesion is malignant, the patient is referred to an oncologic breast surgeon for further evaluation. The patient is then given the options to complete a combined oncoplastic excision or mastectomy, if necessary, with immediate breast reconstruction.

The use of MRI in secondary mastopexy augmentation has not been described previously in the literature. Colombo et al have described the use of MRI in breast reduction surgery which determined that the primary blood supply to the NAC arises from the superomedial intercostal perforators arising from the pectoralis major. Although this may be the predominant blood supply, a significant number of breast reductions have been accomplished based on an inferior pedicle or possibly central mound that may have disrupted this superomedial blood supply. For the plastic and reconstructive surgeon taking on complex secondary mastopexy augmentation procedures or oncoplastic reconstruction cases, the remaining blood supply to the NAC can simply be unknown. The breast MRI with contrast gives the aesthetic and reconstructive surgeon, more information to safely perform these operations and with more confidence. Although we find our method to be useful, we understand that there are several limitations. This is not a randomized controlled trial and is a case review, and furthermore, there may be other more experience surgeons who can perform secondary mastopexy without the use of MRI. However, we find our method to be quite helpful to the plastic and reconstructive surgeon who is well trained, well read, and who chooses to take on the complex cases presented in revision aesthetic and reconstructive surgery.

## CONCLUSIONS

We hope this article serves as a step-by-step template for young plastic and reconstructive surgeons or oncoplastic breast surgeons who choose to take on these difficult cases. Breast MRI with contrast can provide invaluable data that allow safer elevation of the NAC which minimizes complications in secondary mastopexy augmentation cases. MRI also serves as a unique opportunity for early detection of breast cancer. We see MRI as an indispensable aid to preoperative planning and safe NAC preservation.

## Supplementary Material

ojac068_Supplementary_Data

## Notes

Presented at: The Aesthetic Meeting in April 2022, San Diego, CA, USA.
